# A Diabetic Patient Complicated With Pancreatic Cancer After Using Liraglutide: A Case Report

**DOI:** 10.3389/fendo.2020.608966

**Published:** 2021-01-28

**Authors:** Shengjie Wu, Jiabing Wang, Li Jing, Liping Chen

**Affiliations:** ^1^ Department of Pharmacy, Sir Run Run Shaw Hospital, School of Medicine, Zhejiang University, Hangzhou, China; ^2^ Department of Pharmacy, Taizhou Municipal Hospital, Taizhou, China; ^3^ Department of Pharmacy, Nanjing First Hospital, Nanjing Medical University, Nanjing, China

**Keywords:** glucagon-like peptide-1 (GLP-1), glucose-dependent insulinotrophic polypeptide (GIP), liraglutide, pancreatic cancer, diabetes mellitus

## Abstract

**Background:**

Diabetes and cancer are both multifactorial diseases, and epidemiologic evidence indicates that diabetes may be associated with the incidence of certain types of cancer. In diabetes the risk of pancreatic cancer is increased significantly. However, whether certain diabetes treatment being related with the risk of pancreatic cancer remains unclear. In this report, we presented a case of pancreatic ductal adenocarcinoma in a diabetic patient in China after being treated with liraglutide, a novel glucagon-like peptide-1 (GLP-1) analog.

**Case report:**

A 71-year-old Han Chinese man who had had a type 2 diabetes for 25 years presented at the endocrinology department with discomfort in the left upper quadrant of abdomen for 10 days. The patient’s vital signs and laboratory findings were unremarkable except for the elevated level of carbohydrate antigen (CA19-9). The upper abdomen routine enhanced computed tomography (CT) scan showed low density of the pancreatic body and tail, and the histopathological result of the pancreatic biopsy samples was pancreatic ductal adenocarcinoma with regional lymph node metastasis. We reviewed his former medical records and found that liraglutide was added to his hypoglycemic treatment regimen 20 months ago. At that time, the level of tumor biomarkers and upper abdomen routine CT were unremarkable. We estimated the causality between liraglutide and pancreatic cancer by the Naranjo Adverse Drug Reaction Probability scale and WHO-Uppsala Monitoring Centre (WHO-UMC) system, and the causality turned out to be possible.

**Conclusion:**

Our report suggests that liraglutide may be related with the genesis and development of pancreatic cancer and also highlights the importance of regular checkups in diabetic patients treated with liraglutide. However, our report has some notable limitations, and further longer-term follow-up trials with larger sample should be conducted in future to assess the causality between liraglutide and pancreatic cancer.

## Introduction

In type 2 diabetes mellitus, the progressive β-cell failure is partly due to the abnormalities in the incretin axis. Deficiency of GLP-1 and resistance to the action of glucose-dependent insulinotrophic polypeptide (GIP) are the main characteristics of the abnormal incretin effect in type 2 diabetes mellitus (1), for GLP-1 and GIP accounting for ~90% of incretin effect (2, 3). GLP-1 deficiency occurs in the natural history of type 2 diabetes, it is rational to restore the falling insulin response with GLP-1 replacement therapy. Since liraglutide (Victoza), a novel GLP-1 analog approved by FDA in 2010, it has been vastly used in the treatment of type 2 diabetes due to its effective impact on glycemic control, weight loss, and fewer major adverse cardiovascular events (MACEs) occurrence (4). The most frequently reported adverse drug reactions of liraglutide are gastrointestinal ones, such as nausea, diarrhea, and vomiting. However, further studies triggered some concerns that long-term usage of GLP-1 analogs have been shown to result in a massive expansion of exocrine and endocrine pancreatic cells with a potential association with pancreatic cancers (5), and liraglutide is one of the widely used GLP-1 analogs. Clinical data and animal studies published so far remains controversial, and the correlation between liraglutide and pancreatic cancer is not clear yet (6).

## Case Presentation

**Figure 1:**
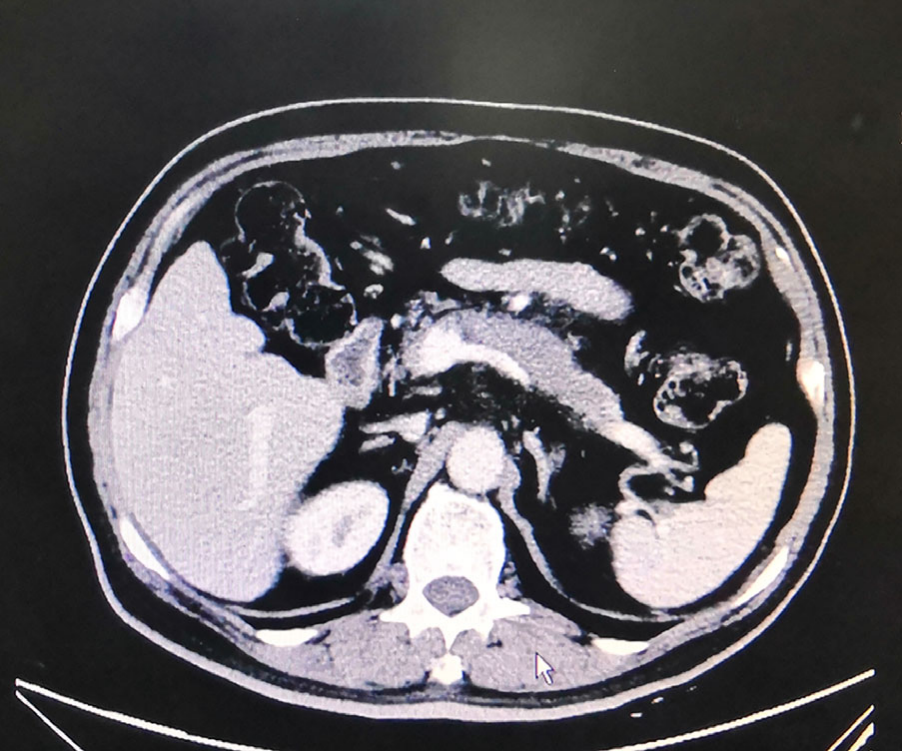
Upper abdomen routine enhanced CT scan of the diabetic patient.

**Figure 2:**
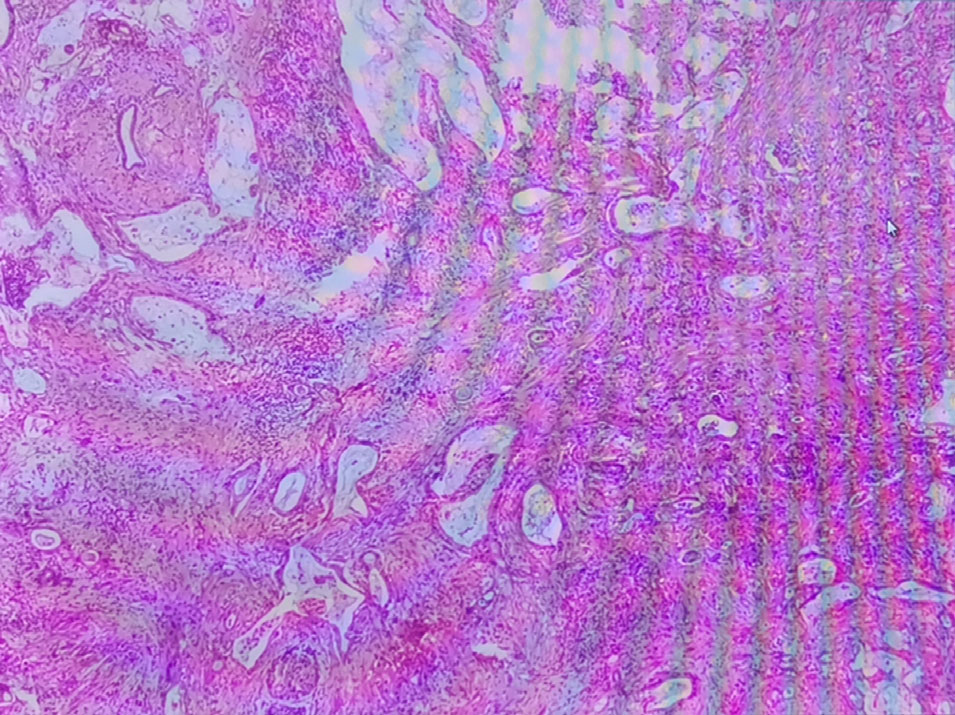
H-E staining of the pancreatic biopsy samples.

We report on a case of pancreatic cancer in association with liraglutide in China. A 71-year-old Han Chinese man who had had a type 2 diabetes for 25 years presented at the endocrinology department with discomfort in the left upper quadrant of abdomen for 10 days. His medical history was significant as it indicated he suffered from coronary atherosclerotic heart disease (CHD), lacunar infarction (LI), and hypertension. His family history was not notable for the presence of diabetes mellitus or hypertension. His psychosocial history was unremarkable either. The patient was an ex-smoker of 10 years ago with a 10-pack year history but never a drinker. Medication included metformin, liraglutide, mixed protamine zinc recombinant human insulin lispro (50R), clopidogrel, and atorvastatin. At the time of admission, the patient’s body mass index (BMI) value was 24.9 kg/m^2^, and his vital signs were unremarkable. Physical examination revealed left upper quadrant tenderness. A blood routine test was within the normal limits. Other laboratory findings showed a blood glucose level of 17.27 mmol/L (3.90–6.10 mmol/L), and a hemoglobin A_1c_ (HbA_1C_) level of 10.8% (4.0–6.0%). The patient’s CA19-9 was >1,000.00 U/ml (<27.00 U/ml), whereas amylase level in blood and urine were unremarkable (**Table 1**). Upper abdomen routine enhanced CT scan showed low density of the pancreatic body and tail and partly coarse wall of splenic artery (**Figure 1**). Regarding that GLP-1 analogs may be involved in the development and progression of pancreatitis or pancreatic cancer, the patient’s antidiabetic regimen was switched from liraglutide to dapagliflozin (10 mg qd po) for his poor glycemic control. After discussing treatment options with the general surgery team, the patient was recommended a CT-guided percutaneous needle biopsy or endoscopic ultrasonography-guided fine needle aspiration (EUS-FNA) of pancreas. On the fourth day of hospitalization, the histopathological result of the pancreatic biopsy samples was pancreatic ductal adenocarcinoma with regional lymph node metastasis (**Figure 2**). He asked for discharge on the fifth day, and unfortunately he has since been lost to follow-up.

**Table 1:** Tumor biomarker values in the case.

Tumor biomarkers	Before using liraglutide	After using liraglutide	Range of normal values
AFP (alpha fetoprotein)	1.63 ng/ml	1.8 ng/ml	0.89–8.78 ng/ml
CEA (carcinoembryonic antigen)	3.95 ng/ml	9.38 ng/ml	<5.00 ng/ml
CA19-9 (carbohydrate antigen)	23 U/ml	>1,000 U/ml	<27.00 U/ml

To investigate the relation between liraglutide and pancreatic cancer, we reviewed the patient’s former medical records in our hospital and found that after early treatment with metformin and mixed protamine zinc recombinant human insulin lispro for more than 5 years, liraglutide (1.2 mg ih qn) was also added to the regimen 20 months ago for his unsatisfactory blood glucose control. He complied with the regimen ever since. We also noticed that before adding liraglutide to antidiabetic regimen, pancreas-related screening was performed in this patient. The level of tumor biomarkers was within the normal limits and the upper abdomen routine CT scan was also unremarkable. We then used the Naranjo Adverse Drug Reaction Probability scale to assess the causality (7), and the score was added up to 3 (**Table 2**) indicating that causality between liraglutide and pancreatic cancer was possible. The causality was reassessed by the WHO-UMC system (8), and still categorized as possible. Therefore, the conclusion could be only drawn that the patient’s pancreatic cancer may be induced by liraglutide.

**Table 2:** ADR probability scale of liraglutide in the case.

	Yes	No	Do not known	Score
1. Are there previous conclusive reports on this reaction?	+1	0	0	1
2. Did the adverse event appear after the suspected drug was administered?	+2	−1	0	2
3. Did the adverse reaction improve when the drug was discontinued or a specific antagonist was administered?	+1	0	0	0
4. Did the adverse reaction reappear when the drug was readministered?	+2	−1	0	0
5. Are there alternative causes (other than the drug) that could on their own have caused the reaction?	−1	+2	0	−1
6. Did the reaction reappear when a placebo was given?	−1	+1	0	0
7. Was the drug detected in the blood (or other fluids) in concentrations known to be toxic?	+1	0	0	0
8. Was the reaction more severe when the dose was increased, or less severe when the dose was decreased?	+1	0	0	0
9. Did the patient have a similar reaction to the same or similar drugs in any previous exposure?	+1	0	0	0
10. Was the adverse event confirmed by any objective evidence?	+1	0	0	1
			Total Score	3

## Discussion and Conclusion

Diabetes and cancer are both multifactorial diseases, and epidemiologic evidence suggests that diabetes is associated with the incidence of certain types of cancer (9). These types of cancer may be induced by the potential effects of hyperglycemia, hyperinsulinemia, and increases in the growth factors or just share certain identical risk factors with diabetes. Ning et al. investigated the risk of 23 common types of cancer among 410,191 patients with type 2 diabetes using the Shanghai Hospital Link database, and the risk of pancreatic cancer was increased significantly (10). Studies also found that certain antidiabetic drugs may be associated with pancreatic cancer. Nowadays, safety concerns of GLP-1 analog therapies being related with pancreatic cancer have been raised. However, evidence from LEADER (Liraglutide Effect and Action in Diabetes: Evaluation of Cardiovascular Outcome Results) cohort indicated that liraglutide was not associated with malignant pancreatic neoplasms. It is worth noting that LEADER was not primarily designed to assess neoplasm risk and firm conclusions cannot be made regarding the numeric imbalances observed for the infrequently occurred pancreatic cancer in individuals (11). To date, evidence from fundamental researches, randomized controlled trials, and real-world studies do not support or refute the association of liraglutide and the increased risk of pancreatic cancer, which still needs to be further studied.

Early in 2017, Talmon et al. reported on a case of GLP-1 analogs induced pancreatic adenocarcinoma in a diabetic patient (12). Whereas in Talmon’s report, the patient was switched from liraglutide to exenatide, and then to glimepiride and insulin before the diagnosis of invasive pancreatic adenocarcinoma was confirmed, which makes the causality assessment complex. Although our case is not the first reported case of pancreatic cancer associated with GLP-1 analogs, it is still necessary to report on this case for not only narrowing down to the liraglutide to blame but highlighting the importance of regular checkups in type 2 diabetic patients treated with liraglutide. Owing to the increased risk of pancreatic cancer in type 2 diabetes mellitus, it will be necessary to fully evaluate the patient’s pancreas condition before started on liraglutide, and to those who have multiple risk factors treated with liraglutide, the regular screening for pancreatic cancer may be particularly important. In practice, asymptomatic, high-risk individuals may derive benefits from surveillance, for pancreatic cancer-specific symptoms occur only at an advanced stage (13). Two former studies used symptoms and CA 19-9 elevation to screen pancreatic cancer in diabetes. Although the prevalence of pancreatic cancer in the screened population was high, most cancers identified were unresectable (14, 15). In our case report, symptoms, tumor biomarkers CA19-9 detection, and upper abdomen routine CT scan provided some clues to the diagnosis of pancreatic cancer, and the EUS-FNA and histopathological testing ultimately confirmed the diagnosis. However, currently we still do not have an optimal screening approach, which may include a unique clinical phenotype, one or more reliable marker(s) of early pancreatic cancer in asymptomatic diabetes and non-invasive imaging with high sensitivity and specificity.

Honestly, the limitations in our report are quite obvious. Smoking habit, alcohol consumption, unsatisfactory blood glucose control, obesity, and chronic pancreatitis are all well-known risk factors in pancreatic cancer (16). In our report, risk factors in the patient include smoking habit, high HbA_1C_ level, overweight, and other medication usage (17). With all these risk factors, the causality between liraglutide and pancreatic cancer can only be categorized as possible. Meanwhile, the development of pancreatic cancer is a multistage process which takes 10 to 15 years (18), 20 months of liraglutide usage in our report seems to be a short time for tumorigenesis. However, Funch et al. found that median days from the initiation of liraglutide to the pancreatic cancer diagnosis were 369 (interquartile range, 226–1,099) (19), and Knapen et al. found that the risk of pancreatic cancer almost doubled in those who had recently initiated GLP-1 analogs therapy (20), which makes the time interval of 20 months plausible. Owing to the relatively low prevalence of pancreatic cancer, this is our first case of pancreatic cancer diagnosed in a diabetic patient treated with liraglutide since 2011 (liraglutide being approved by the National Medical Products Administration of China). This sole case may have some potential bias, and the question whether the liraglutide worked as an independent risk factor in the genesis and development of pancreatic cancer or worked as a triggering factor cannot be answered so far. Therefore, further researches with larger sample on a global scale using the optimal screening approach should be conducted in future to assess whether liraglutide or other GLP-1 analogs are associated with pancreatic cancer or just our “notoriety bias”.

## Data Availability Statement

The original contributions presented in the study are included in the article/supplementary materials. Further inquiries can be directed to the corresponding authors.

## Ethics Statement

Written informed consent was obtained from the individual(s) for the publication of any potentially identifying images or data included in this article.

## Author Contributions

SW and JW participated in the acquisition and analysis of data, as well as writing the manuscript. LC participated in the conceptualization and editing of the manuscript. LJ reviewed the manuscript. All authors contributed to the article and approved the submitted version.

## Funding

This work was supported by the Zhejiang Province Youth Talent Project, part of the Medical and Health Department (2019RC190).

## Conflict of Interest

The authors declare that the research was conducted in the absence of any commercial or financial relationships that could be construed as a potential conflict of interest.
